# Artificial intelligence for detecting temporomandibular joint osteoarthritis using radiographic image data: A systematic review and meta-analysis of diagnostic test accuracy

**DOI:** 10.1371/journal.pone.0288631

**Published:** 2023-07-14

**Authors:** Liang Xu, Jiang Chen, Kaixi Qiu, Feng Yang, Weiliang Wu

**Affiliations:** 1 The School of Stomatology, Fujian Medical University, Fuzhou, Fujian, China; 2 Department of Stomatology, The First Affiliated Hospital of Fujian Medical University, Fuzhou, Fujian, China; 3 School and Hospital of Stomatology, Fujian Medical University, Fuzhou, Fujian, China; 4 Fuzhou No. 1 Hospital Affiliated with Fujian Medical University, Fuzhou, Fujian, China; University of the Pacific Arthur A Dugoni School of Dentistry, UNITED STATES

## Abstract

In this review, we assessed the diagnostic efficiency of artificial intelligence (AI) models in detecting temporomandibular joint osteoarthritis (TMJOA) using radiographic imaging data. Based upon the PRISMA guidelines, a systematic review of studies published between January 2010 and January 2023 was conducted using PubMed, Web of Science, Scopus, and Embase. Articles on the accuracy of AI to detect TMJOA or degenerative changes by radiographic imaging were selected. The characteristics and diagnostic information of each article were extracted. The quality of studies was assessed by the QUADAS-2 tool. Pooled data for sensitivity, specificity, and summary receiver operating characteristic curve (SROC) were calculated. Of 513 records identified through a database search, six met the inclusion criteria and were collected. The pooled sensitivity, specificity, and area under the curve (AUC) were 80%, 90%, and 92%, respectively. Substantial heterogeneity between AI models mainly arose from imaging modality, ethnicity, sex, techniques of AI, and sample size. This article confirmed AI models have enormous potential for diagnosing TMJOA automatically through radiographic imaging. Therefore, AI models appear to have enormous potential to diagnose TMJOA automatically using radiographic images. However, further studies are needed to evaluate AI more thoroughly.

## Introduction

Temporomandibular joint osteoarthritis (TMJOA), a severe subtype of temporomandibular joint disorder, is characterized by progressive absorption of articular cartilage, remodeling of the subchondral bone, and chronic pain [1]. The global incidence of TMJOA is reported to be 8% to 16% [2–4]. The disease severely affects patients’ quality of life, causing excruciating pain and imposing a heavy social and economic burden on individuals and families [5]. Therefore, the importance of early TMJOA diagnosis lies in its potential to enhance treatment efficacy, alleviate symptoms, implement preventive measures, preserve joint function, and optimize healthcare resource utilization. Timely identification and intervention can significantly improve patient outcomes and quality of life while potentially reducing the burden of more invasive and costly treatments.

Presently, the diagnosis of osteoarthritis mainly depends on medical history, disease characteristics, and digital imaging. However, the first two methods sometimes provide limited information about the joint status of patients with TMJOA. Therefore, medical imaging, such as magnetic resonance imaging (MRI), cone-beam computed tomography (CBCT), and orthopantomogram (OPG), is often necessary to assess osteoarthritis [6–8]. The X-ray passed through the temporomandibular joint area and was subsequently detected by a detector. The attenuated X-ray signal is converted into an electrical signal, which is then converted into computed tomography (CT) and OPG images. The principle of nuclear magnetic resonance is what MRI uses. Radio frequency excites nuclei within an external magnetic field to produce a signal and convert it into a medical image. The clinician can detect the morphological changes of bone components in the TMJ with OPG, MRI, and CBCT images.

The above-mentioned noninvasive imaging modalities have been widely used in diagnosing TMJOA [9–11]. Studies have indicated that OPG can only detect apparent erosion, sclerosis, and osteophytes [12]. Although OPG is not sensitive enough to TMJOA [13], it is still an effective tool for the preliminary screening of TMJ. MRI and CBCT were selected to carefully evaluate temporomandibular joint structure because they can identify osseous changes such as erosion, osteophytes, and sclerosis [14]. CBCT is the preferred method of bone evaluation compared to MRI and performs better in diagnosing TMJ osteoarthritis [15]. MRI is also frequently used in TMJOA studies because of advantages in bone evaluation [16]. It not only assesses the condyle morphology but also identifies abnormalities in the bone marrow. Nevertheless, even with such rapid advances in radiological imaging, accurate diagnosis of TMJOA remains challenging. The diagnosis of TMJOA is particularly complex because of the diverse morphological changes of the condyle, such as erosion, flattening, sclerosis, osteophyte, and subcortical cyst [17]. As the diagnostic accuracy of degenerative changes in the condyle depends largely on the radiologists’ expertise and quality of the scanner itself, the interpretation process is susceptible to both misdiagnosis and missed diagnosis [18]. Studies have shown that CBCT has higher accuracy in diagnosing degenerative bone changes than MRI and OPG, with a sensitivity of 0.7–0.9 [19].

Additionally, improvements in technology to extract more accurate and meaningful diagnostic information have made physicians’ work more complex. Therefore, rapid and accurate diagnosis of TMJOA has gradually become a research hotspot.

AI is becoming increasingly popular, especially deep learning (DL), owing to remarkable data mining and processing progress. It is considered a reliable method for combining clinical data and physician reports from electronic medical records to improve the accuracy of various medical tasks [20]. AI has been applied to segment or diagnose lesions automatically in medical images and can be combined with the results of other medical tests to determine disease prognosis [21–23]. In this review, we aimed to assess the diagnostic efficiency of AI models in detecting TMJOA using radiographic image data.

## Materials and methods

Our study protocol was registered on PROSPERO (CRD42023396713). This systematic review and meta-analysis were completed following the Preferred Reporting Items for Systematic Review and Meta-Analyses (PRISMA) guidelines, including search strategy, eligibility criteria, data extraction, risk of bias assessment, and data analysis (S1 Checklist). In addition, the QUADAS-2 tool [24] was used to assess the quality of the included studies.

### Search strategy

Two authors (QKX and WWL) separately performed PubMed, Web of Science, Scopus, and Embase database search using standard search formulas. A comprehensive search was conducted across four databases using a combination of predetermined keywords. The keywords used for the search were ("artificial intelligence" [Mesh] OR "machine learning" [Title/Abstract] OR "neural networks, computer" [Title/Abstract] OR "deep learning" [Title/Abstract]) AND ("temporomandibular joint"[Title/Abstract] OR "osteoarthritis"[Title/Abstract]) AND "sensitivity and specificity"[Title/Abstract]. Additional information regarding the search strategy can be found in the S1 File. Additional articles were identified by manual search. Then, a full-text review was conducted to determine whether the identified literature met the inclusion criteria. If there was any disagreement during the search process, it was settled via consultation with the third author (YF).

### Eligibility criteria

The literature published in PubMed, Web of Science, Scopus, and Embase between January 2010 and January 2023 on the detection of TMJOA using AI in radiographic images was included. There were no restrictions on the countries where the studies were conducted, but only articles published in English were included. The inclusion criteria according to PICOS was as follows: P, patient with TMJOA or degenerative change of condyle; I, AI models including deep learning, machine learning, and radiomic; C, not appliable; O, sensitivity, specificity, AUC value; S, prospective or retrospective study. Excluded criteria were narrative reviews, letters, reviews, editorials, protocol studies, guides, systematic reviews, and meta-analyses.

### Data extraction

The following data were extracted from the included literature: types of AI models, research characteristics, and outcome measurements. To obtain diagnostic accuracy data, a 2×2 confusion matrix, sensitivity, specificity, accuracy, true positive, false positive, true negative, false negative, and the area under the receiver operating characteristic curve (AUROC) were extracted or reconstructed. For further analysis, the following data were extracted: authors, publication year, country, sex, study style, imaging modality, total images, the sample size of test data, and techniques.

### Quality assessment and publication bias

Two independent reviewers (QKX and YF) performed quality assessments of selected studies using the QUADAS-2 criteria. When there was a disagreement, the third author made the final decision based on the criteria. Publication bias was assessed by funnel plot of diagnostic AUC. Asymmetric shape of the funnel plot of included studies indicated study heterogeneity.

### Data analysis

Sensitivity and specificity were calculated by true positives, false positives, true negatives, and false negatives. Forest plots of sensitivity and specificity and a summary receiver operating characteristic (SROC) curve were generated using Stata 15.1. Meta-regression analysis was conducted to estimate the source of heterogeneity when I^2^ was ≥ 50%.

## Results

### Search results

**Fig 1:**
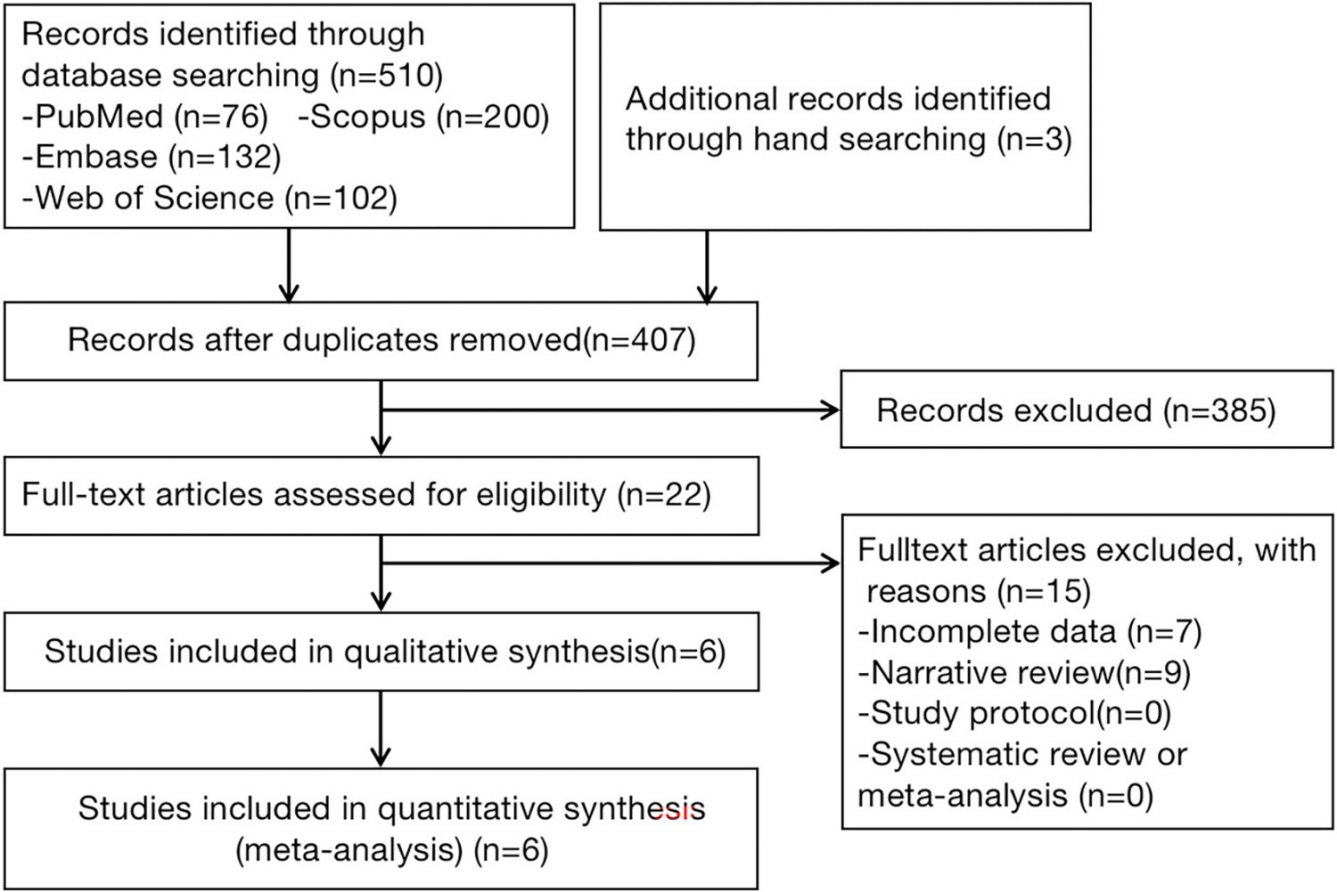
PRISMA flowchart of the included articles.

We retrieved 510 articles from the four databases. Three additional articles were identified through manual screening. After removing 103 duplicates, we analyzed the titles and abstracts of the remaining 407 articles. Twenty-two articles of interest were identified according to the inclusion and exclusion criteria. Sixteen of these articles were subsequently excluded due to incomplete data or a narrative review style. Finally, six studies [25–30] were included in the systematic review. The literature screening process is shown in Fig 1.

### Study characteristics

All six included studies were retrospective (Table 1). MRI was used in one study, CBCT in two studies, and OPG in three studies. Five studies used the Diagnostic Criteria for Temporomandibular Disorders published by Schiffman as the reference standard [31], whereas one study did not offer a reference standard. The research was completed in Belgium, Iran, and Korea, with 66.6% (4/6) of the studies conducted in South Korea. The selected studies utilized one of two AIs: KNN (2/6, 33.3%) or convolutional neural networks (CNN) (4/6, 66.7%).

**Table 1:** Characteristics of individual studies.

author	year	country	study style	total images	Sample size of test data	age and gender	AI Techniques	Female in sample (%)
Eunhye Choi	2021	Korea	retrospective study	1599	272	210males, 979females; mean ± SD age, 37.1 ± 16.0y;	Keras ResNet (CNN)	82.3
K.S. Lee	2020	Korea	retrospective study	3514	300	84 males, 230 females; mean ± SD age, 39.5 ± 18.2y;	single-shot detector (SSD)(CNN)	73.2
Won Jung	2021	Korea	retrospective study	858	172	142 males, 376 females; mean ± SD age, 47.3 ± 20.1y;	EfficientNet-B7 (CNN)	72.6
Donghyun Kim	2020	Korea	retrospective study	1932	231	700 male and 592 female; mean age: 43.3 years	Fine-tuned VGG16 network(CNN)	45.8
Kaan Orhan	2021	Belgium	retrospective study	856	18	34 male and 73 female; mean age: 38 years ± 17.97;	k-nearest neighbors (KNN)	68.2
Haghnegahdar A	2018	Iran	retrospective study	264	264	unclear	k-nearest neighbors (KNN)	unclear

MRI, magnetic resonance imaging; CBCT, cone-beam computed tomography; OPG, orthopantomogram; TMJOA, temporomandibular joint osteoarthritis; CNN, convoluted neural networks; KNN, k-nearest neighbor

### Quality assessment and publication bias

**Fig 2:**
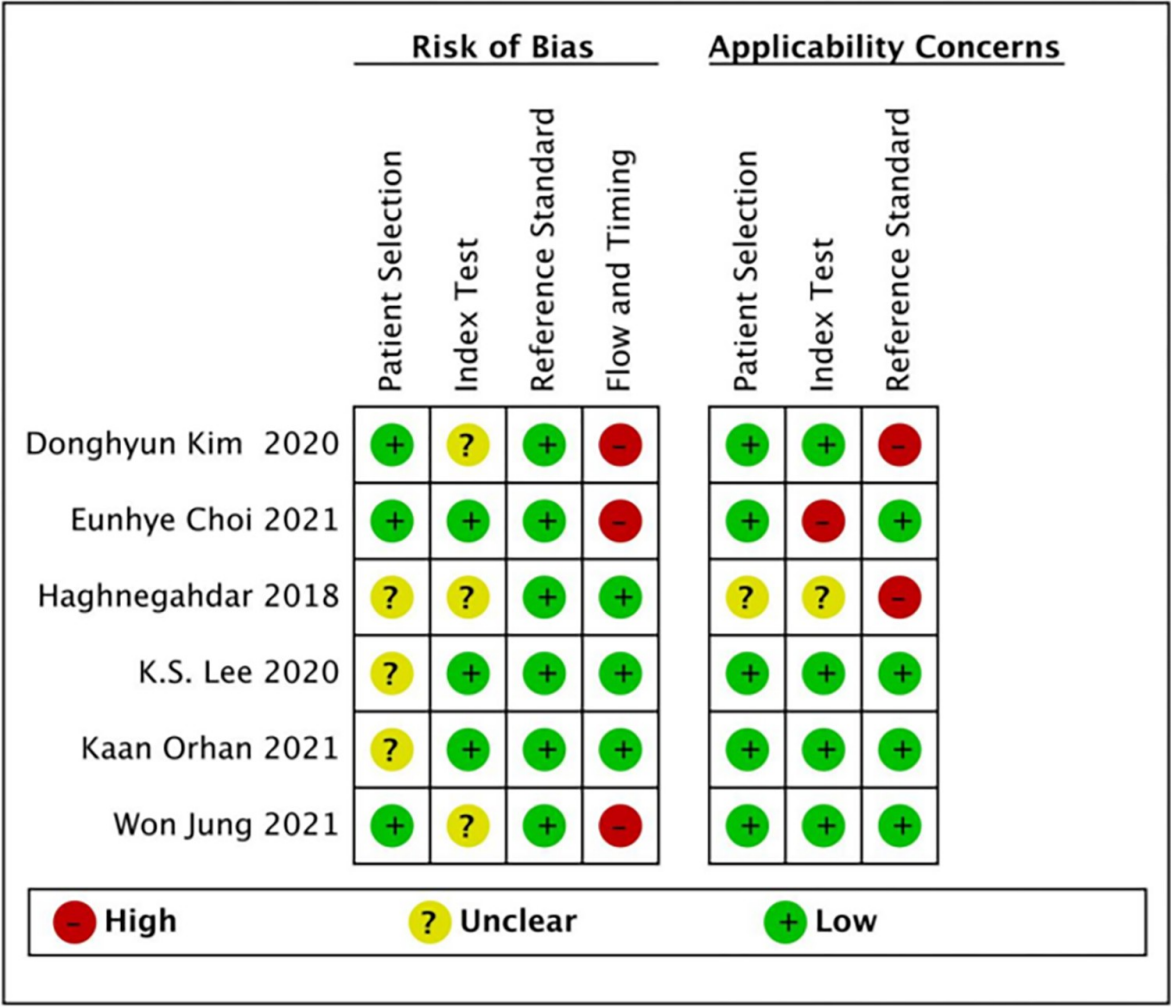
Quality assessment by QUADAS-2 tool.

**Fig 3:**
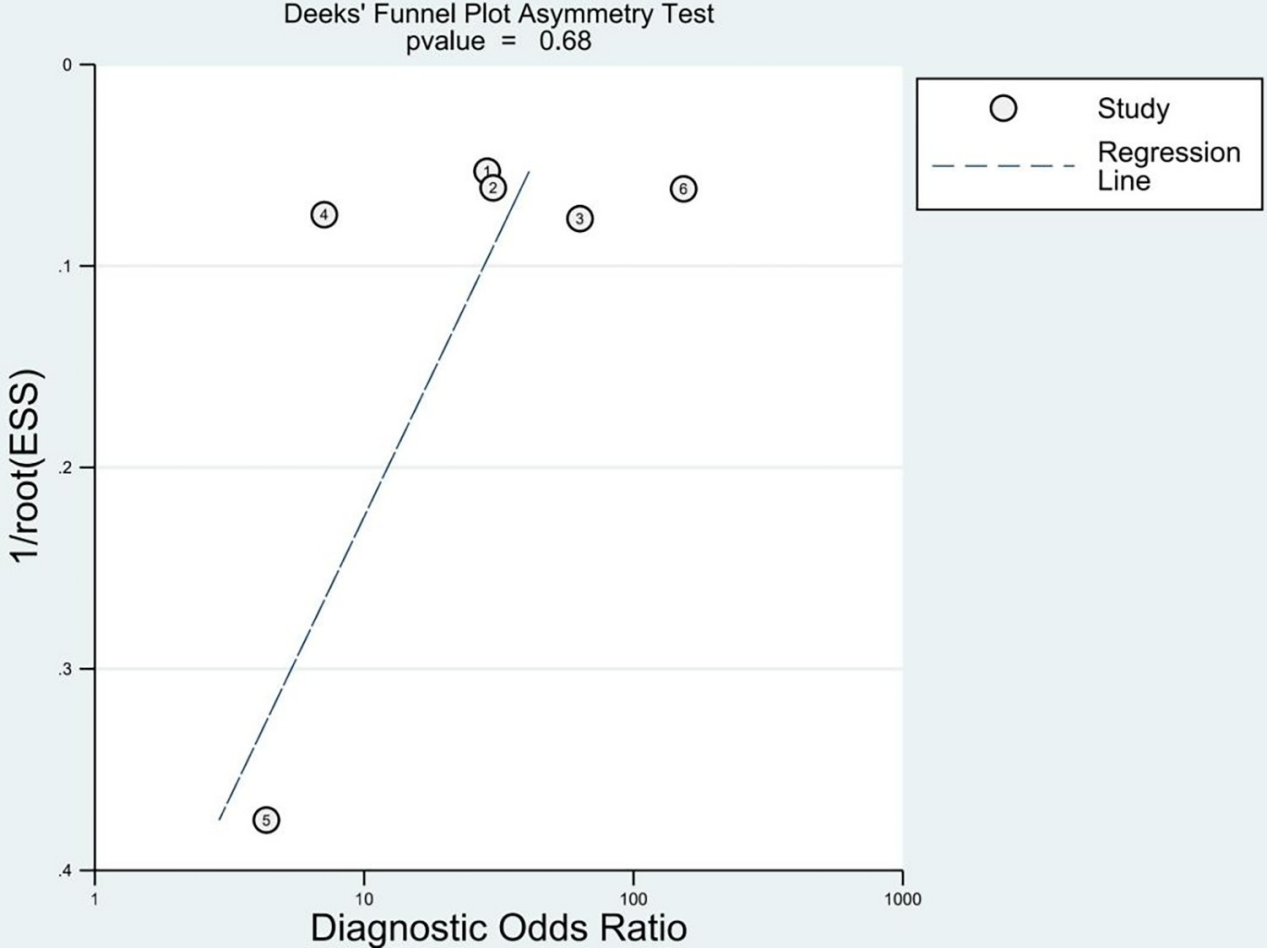
Funnel plot for diagnostic accuracy of AI in detection of TMJOA. TMJOA, temporomandibular joint osteoarthritis; AI, artificial intelligence.

To evaluate the quality of the studies, we applied the QUADAS-2 risk checklist to test the bias risk in each study (Fig 2). The risk of bias in patient selection was low in one half (3/6, 50%) of the studies and unclear in the other half. The same was true for the risk of bias in the index test, flow, and timing. The reference standard test included all six studies with a low risk of bias (100%). Applicability concerns regarding patient selection were low in five (83.3%) studies and unclear in one (16.7%). Applicability concerns in the index test were low in one study (16.7%), high in one study (16.7%), and unclear in four studies (66.6%). Applicability concerns in the reference standard were low in four (66.7%) and high in two (33.3%) studies. The funnel plot assessment (Fig 3) showed no significant publication bias (P = 0.68).

### Diagnostic accuracy

**Fig 4:**
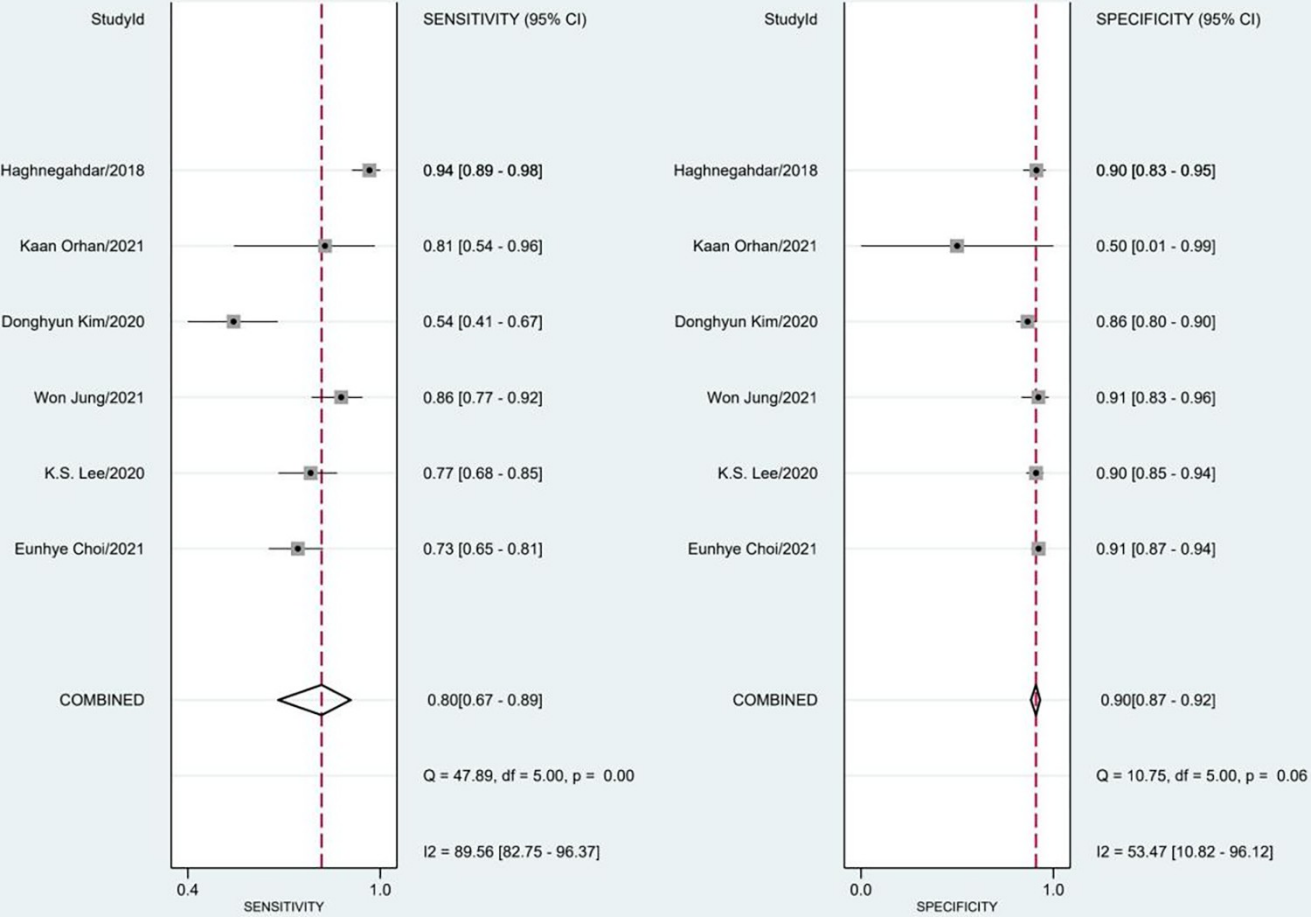
Meta-analysis of sensitivity and specificity for AI.

**Fig 5:**
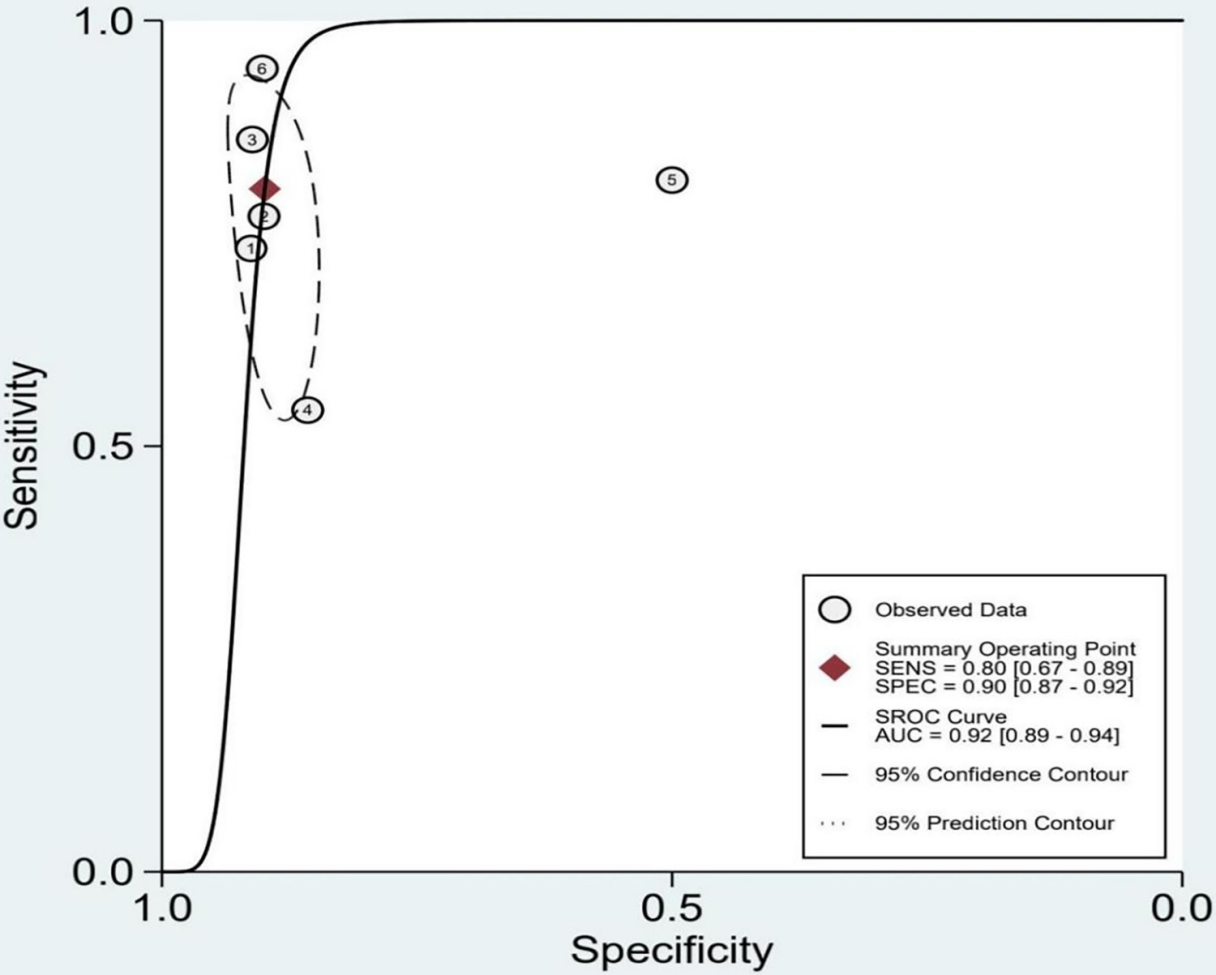
SROC for diagnostic accuracy of AI in detection of TMJOA.

The included studys’ sensitivity and specificity ranged from 0.54 to 0.94 and from 0.50 to 0.91, respectively. The pooled sensitivity and specificity for AI models were 0.80 (95% confidence interval [CI]: 0.67–0.89) with severe heterogeneity (89%) and 0.90 (95% CI: 0.87–0.92) with moderate heterogeneity (53%) (Fig 4). According to the SROC curve, the AUC was 0.92 (95% CI: 0.89–0.94) (Fig 5). We performed a meta-regression analysis to explore the sources of heterogeneity and found that imaging modality, ethnicity, sex, age, AI techniques, and sample size were all possible causes of heterogeneity (Table 2).

**Table 2:** Meta-regression analysis for diagnostic accuracy of AI in detection of TMJOA.

Covariate/Subgroup	Studies(n)	Sensitivity (95%CI)	P-value	Specificity (95%CI)	P-value
Image modality			0.86		<0.001
OPG	3	0.73 (0.58–0.88)		0.90 (0.87–0.93)	
The rest	3	0.87 (0.77–0.97)		0.90 (0.86–0.94)	
Number of total images			<0.001		<0.001
>1000	3	0.69 (0.59–0.80)		0.89 (0.86–0.92)	
≤1000	3	0.90 (0.84–0.96)		0.90 (0.85–0.94)	
Number of test data					
>200	4	0.78 (0.65–0.91)	<0.001	0.90 (0.87–0.92)	<0.001
≤200	2	0.85 (0.69–1.00)		0.90 (0.83–0.97)	
Type of AI techniques					
CNN	4	0.74 (0.63–0.85)	<0.001	0.90 (0.87–0.93)	<0.001
KNN	2	0.91 (0.84–0.99)		0.88 (0.81–0.96)	
Mean age			0.2		<0.001
>40	2	0.73 (0.58–0.88)		0.88 (0.84–0.93)	
≤40	3	0.76 (0.64–0.88)		0.91 (0.88–0.94)	
Female in sample (%)			<0.001		<0.001
>65	4	0.79(0.73–0.84)		0.91(0.88–0.93)	
≤65	1	0.54(0.73–0.84)		0.86(0.81–0.91)	

## Discussion

AI has been used to study knee and hip arthritis, mainly for autodetection, classification, and segmentation. Artificial intelligence (AI) has shown promising applications in the diagnosis of temporomandibular arthritis (TMJ arthritis). AI algorithms have been developed to analyze medical imaging data, such as X-rays, CT, and MRI, to aid in the detection and diagnosis of TMJ arthritis. These algorithms can assist in identifying characteristic features, measuring joint space, assessing bone changes, and detecting early signs of arthritis. By training models on large datasets, AI algorithms can learn to distinguish healthy TMJ joints from those affected by arthritis, enabling more accurate and efficient diagnosis. It is important to note that while AI shows promise in TMJ arthritis diagnosis, its clinical application is still evolving. Further research, validation, and refinement of AI models are needed to ensure their accuracy, reliability, and integration into routine clinical practice. To our knowledge, only a few studies and meta-analyses for the auto-detection of TMJOA have been conducted on temporomandibular osteoarthritis. de Dumast et al. constructed a neural network algorithm to classify TMJOA based on an imaging dataset [32]. Ribera et al. also designed a similar deep learning model [33]. However, the diagnostic accuracy differed appreciably between these studies, ranging from 78.0% to 92.4%. We have retrieved two meta-analyses that bear similarities to our current article. The first study evaluated TMD [34], while the second investigated TMJOA [35]. It is noteworthy that both studies included additional diagnostic biomarkers beyond medical images to establish the diagnosis of TMD and TMJOA. This limitation reduced the scientific value of these two meta-analyses. The present systematic review and meta-analysis aimed to explore the diagnostic rate of AI models developed for the detection of TMJOA on medical images. Six articles were included in our research, comprising 523 images with osteoarthritis and 734 images from controls. After the synthesis, the pooled specificity, sensitivity, and accuracy were 0.80, 0.90, and 0.92, respectively. This high-pooled DTA shows that AI models can successfully differentiate between patients with and without degenerative changes. Researchers have confirmed a similar conclusion in other fields that artificial intelligence is more accurate and reliable than radiologists [36].

The sensitivity of DL in Kim’s literature was only 0.54. We carefully reviewed the literature and found that although model 1 and model 2 could identify condyle region and morphology, respectively, their detection efficiency was relatively low. Adjusting the hyperparameters of the model may result in the desired performance. In Orhans’s literature, the low specificity may be due to insufficient samples in the test set, which is much lower than in other articles. The MRI images in this article were used to analyze osseous changes and disc displacement. Therefore, the samples were selected from patients with temporomandibular joint disorder. The test set’s samples were insufficient because the proportion of TMJOA patients in the total sample was low. However, we agree with the authors on the design of the deep learning model. Therefore, these two articles were eventually included in our meta-analysis.

In our analysis, heterogeneity was observed among the included studies. Therefore, we performed a meta-regression analysis to identify the source of heterogeneity and revealed that imaging modality, ethnicity, sex, age, AI techniques, and sample size might account for heterogeneity. OPG (50.0%), CBCT (33.3%), and MRI (16.7%) were selected as the study images in the included literature. Imaging modalities may be the primary source of heterogeneity. It is generally believed that CBCT is superior to other imaging modalities for diagnosing TMJOA [16]. In our meta-analysis, the included articles based on OPG were less accurate (78%–88%) than those based on CBCT (86%–92%). This is similar to the conclusion from Kaimal S [37], who compared the diagnostic effects of OPG, MRI, and CBCT for TMJOA. Their results showed that the inter-observer reliabilities based on OPG, MRI, and CBCT had a Kappa of 0.16, 0.47, and 0.71, respectively. OPG performance can be poor because the condyles may overlap with the articular eminence or cervical vertebrae in OPG images. In the process of deep learning, the pixels of the overlapping region increase, thus affecting the calculation results. However, CBCT does not have a similar situation because of its three-dimensional nature. Since the included literature using MRI was insufficient, this paper does not discuss it here. The diagnostic efficiency of MRI was inferior to that of CBCT, which was similar to the results of previous studies [16]. The short relaxation time of hard tissue resulted in poor MRI performance [38]. However, many articles still use MRI to study TMJOA [39, 40]. The advantage of MRI is that it can evaluate hard and soft tissues simultaneously to analyze TMJ more comprehensively.

Sample sizes (P < 0.001), including the size of the test data and techniques used to train deep learning (P < 0.001), were also identified as sources of heterogeneity. In general, the performance of deep learning models improves as the amount of data increases [41, 42]. Several experiments have used training sets of different sizes to train deep learning models to determine the ideal sample size [43, 44]. Their results revealed that insufficient sample sizes lead to undertraining, which can affect the final accuracy. A similar situation was observed in this study. The accuracy fluctuated greatly (78%–92%) in the articles with small total sample sizes, while it fluctuated slightly (78%–84%) in the articles with large total sample sizes. The influence of the size of the test data on accuracy was also similar.

Additionally, the performance of deep learning is directly related to the inherent characteristics of deep learning [45, 46]. The included studies constructed two deep learning models: CNN and KNN. The accuracy of CNN was higher than that of KNN, which may be due to their different structures and characteristics. CNN is essentially a mathematical model whose structure and operation logic refer to a biological nervous system, so information can be distributed and processed in parallel [47]. The KNN algorithm, also known as KNN or K-NN, is a supervised learning model for classification [48]. KNN classifies or predicts groupings of individual data points by proximity. Owing to KNN’s relatively simple architecture and algorithm of the KNN, small sample sizes and sample imbalance would lead to classification bias.

In contrast, for larger sample sizes, CNN has better classification ability than KNN. Early studies indicate that the average recognition rate of neural networks is higher than that of the KNN classification method [49, 50]. Two studies in our meta-analysis used small samples to train KNN. Combined with the influence of unbalanced samples, the accuracy of KNN may be lower than that of CNN. This suggests that the architecture of deep learning affects performance. Therefore, different deep-learning algorithms should be compared for different application scenarios to determine the most appropriate one.

Although the prevalence of degenerative joint disorder varies widely [51], the incidence of TMJOA is closely correlated with sex and age. Incidence and severity are higher in women [52]. TMJOA is at least twice as common in women than men [53]. Cellular sexual dimorphism, hormones, genetic factors, and immune modulation mechanisms may contribute to the sex disparity observed in TMJOA [54–56]. Numerous studies have confirmed the strong correlation between age and TMJOA [57, 58]. TMJOA has significantly different peak age characteristics, which are approximately 30 and 55 years [59]. This relationship reflects the inherent accumulation of tissue damage owing to a gradual decline in cellular adaptation.

Although the title of our search include artificial intelligence, almost all the literature included in the research used deep learning technology. Deep learning does not require artificial feature sets compared to traditional machine learning. Feature extraction is data-driven, which enables deep learning to extract deeper features. Meanwhile, higher precision and stronger robustness make deep learning more widely used. Nevertheless, deep learning has its limits. First, deep learning does not negate any data nor detect hidden biases in the data, which can lead to unobjective results. Second, deep learning is susceptible to counterattack, which leads to radically different judgments. Third, it can only find correlations between events but cannot explain causation. Finally, the performance of deep learning depends on the size of the data set, which requires high computational power. Despite these problems, deep learning is still a promising tool.

This meta-analysis only included six papers, which may lead to the need for more objective conclusions. However, this is because the research on deep learning in temporomandibular osteoarthritis is still exploratory, and more literature meeting the inclusion criteria are needed. For this reason, we expanded the search scope to include LILACS, Scopus, Web of Science, and other databases, but no other literature meeting the inclusion criteria was retrieved. We will regularly update this paper in the later stages to ensure its timeliness.

## Conclusion

AI models appear to have enormous potential to diagnose TMJOA automatically using radiographic images. Although AI still has shortcomings in automatic diagnosis and various models differ in accuracy, its high average accuracy still makes it an auxiliary means to avoid misdiagnosis or missed diagnosis. However, further studies are needed to evaluate AI more thoroughly.

## Supporting information

S1 Checklist PRISMA checklist. (PDF)

S1 File Search strategy. (DOCX)
