# Supplementary material for: Artificial intelligence for detecting temporomandibular joint osteoarthritis using radiographic image data: A systematic review and meta-analysis of diagnostic test accuracy
Source: PLoS One. 2023 Jul 14;18(7):e0288631. doi: 10.1371/journal.pone.0288631 (PMC10348514; doi:10.1371/journal.pone.0288631)
Supplement: S1 File — (DOCX) [file pone.0288631.s002.docx]

Supplementary Table 1 Search strategy in PubMed, Embase, Web of Science and Scopus.

| Database | Search strategy |
| --- | --- |
| PubMed | ("deep learning"[Title/Abstract] OR "learning deep"[Title/Abstract] OR "hierarchical learning"[Title/Abstract] OR "learning hierarchical"[Title/Abstract] OR ("artificial intelligence"[Title/Abstract] OR "intelligence artificial"[Title/Abstract] OR "computational intelligence"[Title/Abstract] OR "intelligence computational"[Title/Abstract] OR "machine intelligence"[Title/Abstract] OR "intelligence machine"[Title/Abstract] OR "computer reasoning"[Title/Abstract] OR "reasoning computer"[Title/Abstract] OR (("antagonists and inhibitors"[MeSH Subheading] OR ("antagonists"[All Fields] AND "inhibitors"[All Fields]) OR "antagonists and inhibitors"[All Fields] OR "ai"[All Fields]) AND "artificial intelligence"[Title/Abstract]) OR "computer vision systems"[Title/Abstract] OR "computer vision system"[Title/Abstract] OR "system computer vision"[Title/Abstract] OR "systems computer vision"[Title/Abstract] OR (("vision s"[All Fields] OR "vision, ocular"[MeSH Terms] OR ("Vision"[All Fields] AND "ocular"[All Fields]) OR "ocular vision"[All Fields] OR "Vision"[All Fields] OR "visions"[All Fields] OR "visioning"[All Fields]) AND "system computer"[Title/Abstract]) OR (("vision s"[All Fields] OR "vision, ocular"[MeSH Terms] OR ("Vision"[All Fields] AND "ocular"[All Fields]) OR "ocular vision"[All Fields] OR "Vision"[All Fields] OR "visions"[All Fields] OR "visioning"[All Fields]) AND "systems computer"[Title/Abstract]) OR (("education"[MeSH Terms] OR "education"[All Fields] OR ("knowledge"[All Fields] AND "acquisition"[All Fields]) OR "knowledge acquisition"[All Fields]) AND "Computer"[Title/Abstract]) OR (("acquisition"[All Fields] OR "acquisitions"[All Fields]) AND ("knowledge"[MeSH Terms] OR "knowledge"[All Fields] OR "knowledge s"[All Fields] OR "knowledgeability"[All Fields] OR "knowledgeable"[All Fields] OR "knowledgeably"[All Fields] OR "knowledges"[All Fields]) AND "Computer"[Title/Abstract]) OR (("knowledge"[MeSH Terms] OR "knowledge"[All Fields] OR "knowledge s"[All Fields] OR "knowledgeability"[All Fields] OR "knowledgeable"[All Fields] OR "knowledgeably"[All Fields] OR "knowledges"[All Fields]) AND ("representability"[All Fields] OR "representable"[All Fields] OR "representation"[All Fields] OR "representation s"[All Fields] OR "representational"[All Fields] OR "representations"[All Fields]) AND "Computer"[Title/Abstract]) OR (("knowledge"[MeSH Terms] OR "knowledge"[All Fields] OR "knowledge s"[All Fields] OR "knowledgeability"[All Fields] OR "knowledgeable"[All Fields] OR "knowledgeably"[All Fields] OR "knowledges"[All Fields]) AND ("representability"[All Fields] OR "representable"[All Fields] OR "representation"[All Fields] OR "representation s"[All Fields] OR "representational"[All Fields] OR "representations"[All Fields]) AND "Computer"[Title/Abstract]) OR (("representability"[All Fields] OR "representable"[All Fields] OR "representation"[All Fields] OR "representation s"[All Fields] OR "representational"[All Fields] OR "representations"[All Fields]) AND ("knowledge"[MeSH Terms] OR "knowledge"[All Fields] OR "knowledge s"[All Fields] OR "knowledgeability"[All Fields] OR "knowledgeable"[All Fields] OR "knowledgeably"[All Fields] OR "knowledges"[All Fields]) AND "Computer"[Title/Abstract])) OR ("machine learning"[Title/Abstract] OR "learning machine"[Title/Abstract] OR "transfer learning"[Title/Abstract] OR "learning transfer"[Title/Abstract]) OR ("neural networks computer"[Title/Abstract] OR "computer neural network"[Title/Abstract] OR "computer neural networks"[Title/Abstract] OR (("Network"[All Fields] OR "network s"[All Fields] OR "networked"[All Fields] OR "networker"[All Fields] OR "networkers"[All Fields] OR "networking"[All Fields] OR "Networks"[All Fields]) AND "computer neural"[Title/Abstract]) OR (("Network"[All Fields] OR "network s"[All Fields] OR "networked"[All Fields] OR "networker"[All Fields] OR "networkers"[All Fields] OR "networking"[All Fields] OR "Networks"[All Fields]) AND "computer neural"[Title/Abstract]) OR "neural network computer"[Title/Abstract] OR "models neural network"[Title/Abstract] OR "model neural network"[Title/Abstract] OR "network model neural"[Title/Abstract] OR (("Network"[All Fields] OR "network s"[All Fields] OR "networked"[All Fields] OR "networker"[All Fields] OR "networkers"[All Fields] OR "networking"[All Fields] OR "Networks"[All Fields]) AND "models neural"[Title/Abstract]) OR "neural network model"[Title/Abstract] OR "neural network models"[Title/Abstract] OR "computational neural networks"[Title/Abstract] OR "computational neural network"[Title/Abstract] OR (("Network"[All Fields] OR "network s"[All Fields] OR "networked"[All Fields] OR "networker"[All Fields] OR "networkers"[All Fields] OR "networking"[All Fields] OR "Networks"[All Fields]) AND "computational neural"[Title/Abstract]) OR "networks computational neural"[Title/Abstract] OR "neural network computational"[Title/Abstract] OR "neural networks computational"[Title/Abstract] OR "Perceptrons"[Title/Abstract] OR "Perceptron"[Title/Abstract] OR "connectionist models"[Title/Abstract] OR "connectionist model"[Title/Abstract] OR "model connectionist"[Title/Abstract] OR "models connectionist"[Title/Abstract] OR (("neural networks, computer"[MeSH Terms] OR ("Neural"[All Fields] AND "Networks"[All Fields] AND "Computer"[All Fields]) OR "computer neural networks"[All Fields] OR ("Neural"[All Fields] AND "Networks"[All Fields]) OR "neural networks"[All Fields]) AND "Computer"[Title/Abstract]) OR (("Network"[All Fields] OR "network s"[All Fields] OR "networked"[All Fields] OR "networker"[All Fields] OR "networkers"[All Fields] OR "networking"[All Fields] OR "Networks"[All Fields]) AND ("Neural"[All Fields] OR "neuralization"[All Fields] OR "neuralize"[All Fields] OR "neuralized"[All Fields] OR "neuralizes"[All Fields] OR "neuralizing"[All Fields] OR "neurally"[All Fields]) AND "Computer"[Title/Abstract]) OR (("neural networks, computer"[MeSH Terms] OR ("Neural"[All Fields] AND "Networks"[All Fields] AND "Computer"[All Fields]) OR "computer neural networks"[All Fields] OR ("Networks"[All Fields] AND "Neural"[All Fields]) OR "networks neural"[All Fields]) AND "Computer"[Title/Abstract]) OR (("neural networks, computer"[MeSH Terms] OR ("Neural"[All Fields] AND "Networks"[All Fields] AND "Computer"[All Fields]) OR "computer neural networks"[All Fields] OR ("Neural"[All Fields] AND "Network"[All Fields]) OR "neural network"[All Fields]) AND "Computer"[Title/Abstract]))) AND ("temporomandibular joint"[Title/Abstract] OR "joint temporomandibular"[Title/Abstract] OR "joints temporomandibular"[Title/Abstract] OR "temporomandibular joints"[Title/Abstract] OR "TMJ"[Title/Abstract] OR ("Osteoarthritis"[Title/Abstract] OR "Osteoarthritides"[Title/Abstract] OR "Osteoarthrosis"[Title/Abstract] OR "Osteoarthroses"[Title/Abstract] OR "arthritis degenerative"[Title/Abstract] OR (("Arthritis"[MeSH Terms] OR "Arthritis"[All Fields] OR "Arthritides"[All Fields] OR "polyarthritides"[All Fields]) AND "Degenerative"[Title/Abstract]) OR "degenerative arthritides"[Title/Abstract] OR "degenerative arthritis"[Title/Abstract] OR "Arthrosis"[Title/Abstract] OR "Arthroses"[Title/Abstract] OR "osteoarthrosis deformans"[Title/Abstract])) AND (("Sensitivity"[Title/Abstract] AND "Specificity"[Title/Abstract]) OR ("Specificity"[Title/Abstract] AND "Sensitivity"[Title/Abstract]) OR "Sensitivity"[Title/Abstract] OR "Specificity"[Title/Abstract]) |
| Embase | ('osteoarthritis':ab,ti OR 'osteoarthritides':ab,ti OR 'osteoarthrosis':ab,ti OR 'osteoarthroses':ab,ti OR 'arthritis, degenerative':ab,ti OR 'arthritides, degenerative':ab,ti OR 'degenerative arthritides':ab,ti OR 'degenerative arthritis':ab,ti OR arthrosis:ab,ti OR arthroses:ab,ti OR 'osteoarthrosis deformans':ab,ti OR 'temporomandibular joint':ab,ti OR 'joint, temporomandibular':ab,ti OR 'joints,temporomandibular':ab,ti OR 'temporomandibular joints':ab,ti OR tmj:ab,ti) AND ('deep learning':ab,ti OR 'learning, deep':ab,ti OR 'hierarchical learning':ab,ti OR 'learning, hierarchical':ab,ti OR 'machine learning':ab,ti OR 'learning, machine':ab,ti OR 'transfer learning':ab,ti OR 'learning, transfer':ab,ti OR 'artificial intelligence':ab,ti OR 'intelligence, artificial':ab,ti OR 'computational intelligence':ab,ti OR 'intelligence, computational':ab,ti OR 'machine intelligence':ab,ti OR 'intelligence, machine':ab,ti OR 'computer reasoning':ab,ti OR 'reasoning, computer':ab,ti OR 'ai artificial intelligence':ab,ti OR 'computer vision systems':ab,ti OR 'computer vision system':ab,ti OR 'system, computer vision':ab,ti OR 'systems, computer vision':ab,ti OR 'vision system, computer':ab,ti OR 'vision systems, computer':ab,ti OR 'knowledge acquisition computer':ab,ti OR 'acquisition, knowledge computer':ab,ti OR 'knowledge representation computer':ab,ti OR 'knowledge representations computer':ab,ti OR 'representation, knowledge computer':ab,ti OR 'neural network':ab,ti OR 'computer neural network':ab,ti OR 'computer neural networks':ab,ti OR 'network, computer neural':ab,ti OR 'networks, computer neural':ab,ti OR 'neural network, computer':ab,ti OR 'models, neural network':ab,ti OR 'model, neural network':ab,ti OR 'network model, neural':ab,ti OR 'network models, neural':ab,ti OR 'neural network model':ab,ti OR 'neural network models':ab,ti OR 'computational neural networks':ab,ti OR 'computational neural network':ab,ti OR 'network, computational neural':ab,ti OR 'networks, computational neural':ab,ti OR 'neural network, computational':ab,ti OR 'neural networks, computational':ab,ti OR perceptrons:ab,ti OR perceptron:ab,ti OR 'connectionist models':ab,ti OR 'connectionist model':ab,ti OR 'model, connectionist':ab,ti OR 'models, connectionist':ab,ti OR 'neural networks computer':ab,ti OR 'network, neural computer':ab,ti OR 'networks, neural computer':ab,ti OR 'neural network computer':ab,ti) AND ('specificity and sensitivity':ab,ti OR 'specificity':ab,ti OR 'sensitivity':ab,ti) |
| Web of Science | TS=((temporomandibular joint OR joint, temporomandibular OR joints, temporomandibular OR temporomandibular joints OR TMJ OR osteoarthritis OR osteoarthritises OR osteoarthrosis OR osteoarthrosis OR arthritis, degenerative OR arthritides, degenerative OR degenerative arthritides OR degenerative arthritis OR arthrosis OR arthroses OR osteoarthrosis deformans) AND (artificial intelligence OR intelligence, artificial OR computational intelligence OR intelligence, computational OR machine intelligence OR intelligence, machine OR computer reasoning OR reasoning, computer OR AI OR computer vision systems OR computer vision system OR system, computer vision OR systems, computer vision OR vision system, computer OR vision systems, computer OR knowledge acquisition (computer) OR acquisition, knowledge (computer) OR knowledge representation (computer) OR knowledge representations (computer) OR representation, knowledge (computer) OR machine learning OR learning, machine OR transfer learning OR learning, transfer OR neural networks, computer OR computer neural network OR computer neural networks OR network, computer neural OR networks, computer neural OR neural network, computer OR models, neural network OR model, neural network OR network model, neural OR network models, neural OR neural network model OR neural network models OR computational neural networks OR computational neural network OR network, computational neural OR networks, computational neural OR neural network, computational OR neural networks, computational OR perceptrons OR perceptron OR connectionist models OR connectionist model OR model, connectionist OR models, connectionist OR neural networks (computer) OR network, neural (computer) OR networks, neural (computer) OR neural network (computer) OR deep learning OR learning, deep OR hierarchical learning OR learning, hierarchical) AND (specificity and sensitivity OR specificity OR sensitivity)) |
| Scopus | (“specificity and sensitivity” OR “specificity” OR “sensitivity”) AND ("temporomandibular joint" OR "joint, temporomandibular" OR "joints, temporomandibular" OR "temporomandibular joints" OR "TMJ") AND ("osteoarthritis" OR "osteoarthritides" OR "osteoarthrosis" OR "osteoarthroses" OR "arthritis, degenerative" OR "arthritides, degenerative" OR "degenerative arthritides" OR "degenerative arthritis" OR "arthrosis" OR "arthroses" OR "osteoarthrosis deformans") AND (("neural networks, computer" OR "computer neural network" OR "computer neural networks" OR "network, computer neural" OR "networks, computer neural" OR "neural network, computer" OR "models, neural network" OR "model, neural network" OR "network model, neural" OR "network models, neural" OR "neural network model" OR "neural network models" OR "computational neural networks" OR "computational neural network" OR "network, computational neural" OR "networks, computational neural" OR "neural network, computational" OR "neural networks, computational" OR "perceptrons" OR "perceptron" OR "connectionist models" OR "connectionist model" OR "model, connectionist" OR "models, connectionist" OR "neural networks (computer)" OR "network, neural (computer)" OR  "networks, neural (computer)" OR "neural network (computer)") OR ("deep learning" OR "learning, deep" OR "hierarchical learning" OR "learning, hierarchical") OR ("machine learning" OR "learning, machine" OR "transfer learning" OR "learning, transfer") OR ("artificial intelligence" OR "intelligence, artificial" OR "computational intelligence" OR "intelligence, computational" OR "machine intelligence" OR "intelligence, machine" OR "computer reasoning" OR "reasoning, computer" OR "AI" OR "computer vision systems" OR "computer vision system" OR "system, computer vision" OR "systems, computer vision" OR "vision system, computer" OR "vision systems, computer" OR "knowledge acquisition (computer)" OR  "acquisition, knowledge (computer)" OR "knowledge representation (computer)" OR "knowledge representations (computer)" OR "representation, knowledge (computer)")) |
